# Characterization of *BRCA1* and *BRCA2* genetic variants in a cohort of Bahraini breast cancer patients using next‐generation sequencing

**DOI:** 10.1002/mgg3.771

**Published:** 2019-05-26

**Authors:** Fatima Al Hannan, Michael B. Keogh, Safa Taha, Latifa Al Buainain

**Affiliations:** ^1^ Department of Basic Medical Sciences Royal College of Surgeons in Ireland Busaiteen Bahrain; ^2^ Al Jawhara Centre for Molecular Medicine & Inherited Disorders Arabian Gulf University Manama Bahrain; ^3^ Department of Surgery Bahrain Defence Force Hospital Manama Bahrain

**Keywords:** Bahrain, *BRCA1/2*, breast cancer, next‐generation sequencing, variants

## Abstract

**Background:**

Breast cancer is the most common malignancy in women worldwide. About 5%–10% are due to hereditary predisposition. The contribution of *BRCA1/2* mutations to familial breast cancer in Bahrain has not been explored. The objective of this study was to investigate the spectrum of *BRCA1/2* genetic variants and estimate their frequencies in familial breast cancer. We also aim to test the efficiency of the next‐generation sequencing (NGS) as a powerful tool for detecting genetic variation within *BRCA1/2* genes.

**Methods:**

Twenty‐five unrelated female patients diagnosed with familial breast cancer were screened for *BRCA1/2* variants. All targeted coding exons and exon–intron boundaries of *BRCA1/2* genes were amplified with 167 pairs of primers by NGS.

**Results:**

We have identified two deleterious *BRCA1/2* variants in two patients, one in *BRCA1* gene (c.4850C>A) and other in *BRCA2* gene (c.67+2T>C). In addition to the deleterious variants, we identified 24 distinct missense variants of uncertain significance, 10 of them are seen to confer minor but cumulatively significant risk of breast cancer.

**Conclusion:**

Our data suggest that *BRCA1/2* variants may contribute to the pathogenesis of familial breast cancer in Bahrain. It also shows that NGS is useful tool for screening *BRCA1/2* genetic variants of probands and unaffected relatives.

## 1 INTRODUCTION

Breast cancer is one of the most common malignancies affecting women worldwide (Ferlay et al., 2015). Approximately, 5%–10% of breast cancer cases may possess breast cancer susceptibility genes predisposing to an increased risk of malignancy (Cobain, Milliron, & Merajver, 2016). Genetic linkage studies have identified *BRCA1* (OMIM# 113705) and BRCA2 (OMIM# 600185) as two major genes associated with hereditary breast cancer and high breast cancer risk. These tumor suppressor genes were reported and identified as breast cancer susceptibility genes for the first time in 1990 and 1995 and are located on chromosome 17q21 and 13q12‐13, respectively (Hall et al., 1990; Wooster et al., 1995). The functions of *BRCA1/2* are crucial for normal cell function as they are involved in the cellular DNA repair and inhibition of uncontrolled cell growth (Lou et al., 2014). Germline mutations in these two highly penetrant genes are inherited in an autosomal dominant pattern and can increase the lifetime risk of developing breast cancer by as much as 80%. Women who carry *BRCA1/2* mutations have a significantly increased risk of developing breast cancer before the age of 50 years (Couch, Nathanson, & Offit, 2014).

More than 1,800 distinct *BRCA1* and 2,000 *BRCA2* mutations have been reported in the Breast Cancer Information Core (BIC) database (Couch et al., 2014). These mutations are widely scattered across both genes and most affect the structure, integrity and function of the gene. Some studies have shown that the proportion of *BRCA1/2* mutations could be higher in Arab women when compared to other populations (Tadmouri, Sastry, & Chouchane, 2014). Studies in Morocco (Jouali et al., 2016; Laraqui et al., 2013), Algeria (Cherbal et al., 2010; Henouda et al., 2016), Tunisia (Mahfoudh et al., 2012; Riahi et al., 2015), Middle East (Bu et al., 2016), Egypt (Kim et al., 2017), Lebanon (Jalkh et al., 2017), Saudi Arabia (Merdad et al., 2015), and Qatar (Bujassoum, Bugrein, & Al‐Sulaiman, 2017) have revealed the presence of *BRCA1/2* mutations in cases of familial breast cancer. In Bahrain, no research has been done so far to elucidate the genetic background of heritable breast cancer although Bahrain has the highest incidence of breast cancers compared to other countries in GCC. The age‐standardized rate per 100,000 women is 53.4 for Bahrain followed by Qatar (48.2) and Kuwait (46.6) (Chouchane, Boussen, & Sastry, 2013). Breast cancer in Bahrain has specific characteristics; the most important is the relatively younger age of onset and advanced stage at presentation (Hamadeh, Abulfatih, Fekri, & Al‐Mehza, 2014). These observations can be attributed to undetermined genetic lesions accumulating in the population due to consanguineous marriages and increased exposure to environmental insults as well as differences in fertility rates and duration of breastfeeding (Hamadeh et al., 2014; Ravichandran & Al‐Zahrani, 2009).

In Bahrain, the breast cancer screening by mammography was introduced in 2005 and essentially subjected to high‐risk suspected cases for women aged 40 and above. The screening program since introduced has contributed to the diagnosis of 12.7% of the cases (Hamadeh et al., 2014). The low percentage of detected cases merits further evaluation of the Bahraini cancer prevention programs to better screening services that aid in early detection and defining of high‐risk individuals in the population. The main objective of the present study was to investigate the contribution of *BRCA1/2* variants in familial and early onset breast cancer in Bahraini women using next‐generation sequencing (NGS) and to test the efficiency of the NGS in the diagnosis and screening programs of breast cancer patients in the region. The overall objective was to better understand the genetic risk factors associated with the disease which could eventually lead to an earlier detection with better prognosis and survival rates.

## 2 MATERIALS AND METHODS

### 2.1 Editorial policies and ethical considerations

The study was conducted in accordance with the Helsinki declaration and the study protocol was approved by the Ethical research committees of Royal college of Surgeons in Ireland‐Bahrain and Bahrain Defence Force hospital in Bahrain. All the participants signed informed consent.

### 2.2 Patients

Patients were selected for this study if they have at least one of the following conditions: early onset breast cancer, at least one first‐ or two‐second degree relatives with breast cancer; breast cancer with advanced tumor staging/grading. Peripheral blood was obtained from 25 different unrelated Bahraini families following obtaining their informed consent and their family history of breast cancer. Patient's recruitment and blood sampling were all performed according to the institutional ethical procedures. All samples were anonymized and blindly sequenced and analyzed.

### 2.3 DNA extraction

Genomic DNA was isolated from 200 µl peripheral blood anti‐coagulated with EDTA on the MagNa Pure LC instrument using MagNa Pure LC DNA Isolation Kit (Roche Diagnostics GmbH, Mannheim), according to the manufacturer's instructions. The concentration of DNA was determined by using the Nano Drop^™^ 2000 spectrophotometer (Thermos Fisher, Yokohama, Japan).

### 2.4 Next‐generation sequencing analysis

All targeted coding exons and exon–intron boundaries of *BRCA1/2* genes were amplified with 167 pairs of primers in three primer pair pools. After the targeted amplification and construction of a library through Ion AmpliSeq^™^ Library Kit 2.0, the nucleotide sequences of the targeted regions are analyzed by Life Technologies Ion PGM platform on an Ion 316 Chip. Sequence variants are identified by Torrent Suite 4.2 (Life Technologies, Tokyo, Japan). The clinical significance of each sequence variant is suggested on the basis of reference to ClinVar and BIC (Landrum et al., 2014). The NGS protocol which we used has been explained elsewhere (Hirotsu et al., 2015).

The reference sequences used were NM_007294.3 for *BRCA1* and NM_000059.3 for *BRCA2*. Minor allele frequency was determined from the 1000 Genomes Project database (Abecasis et al., 2012) http://www.internationalgenome.org/1000-genomes-browsers/ and the Human Genetic Variation Database (http://www.hgvd.genome.med.kyoto-u.ac.jp/).

## 3 RESULTS

### 3.1 Clinical characteristics and detection of *BRCA1/2* variants

To analyse the prevalence of breast cancer with *BRCA1/2* genetic variants, we screened *BRCA1/2* variants in the 25 recruited patients with personal and family history of breast cancer. All women were diagnosed with unilateral breast cancer. The median age at diagnosis of breast cancer was 47 years (range 33–63). DNA was extracted from patient's peripheral blood samples. We amplified targeted coding regions and exon–intron boundaries of *BRCA1/2* genes using multiplex PCR. A total of 167 primer pairs were used in three primer pools covering 16.25 KB of target genomic sequence. Targeted genomic sequencing was performed using the Ion Torrent PGM System from Life Technologies generating short sequence reads of approximately 200 bp. We have identified two deleterious *BRCA1/2* variants in two patients, one in *BRCA1* gene (c.4850C>A) and other in *BRCA2* gene (c.67+2T>C). In addition to the deleterious variants, we have identified 24 missense variants of uncertain significance (VUS).

### 3.2 **Clinical presentation of the heterozygous carrier of the *BRCA1* variant c.4850C**>**A**

The carrier of *BRCA1* variant c.4850C>A was diagnosed with right breast cancer at age 33 and had a positive family history of breast cancer (a mother diagnosed with breast cancer at age 57, a daughter and maternal aunt diagnosed with breast cancer before age 40). There was no family history of ovarian cancer. Patient presented with right breast lump noticed 2 days prior to presentation that radiological investigations did not reveal any breast lesions or distant metastasis; however, fine needle aspiration reported malignancy.

The tumor was classified as a stage 2 loco‐regional malignancy, and the histopathology results confirmed the diagnosis of an invasive ductal carcinoma grade 3, measuring 23 mm in maximum dimension. Two palpable mobile axillary nodes were observed during examination. The patient underwent wide local excision of right breast tumor and right axillary clearance.

### 3.3 Clinical presentation of the heterozygous carrier of the *BRCA2* variant c.67+2T>C

This is a Bahraini woman, known case of hypertension and hyperlipidemia with history of abdominal hysterectomy and bilateral oophorectomy for endometrial cancer 9 years prior to her presentation. The patient has a very strong family history of breast cancer including her mother and maternal aunt (diagnosed before age 50). She has presented with right axillary swelling and right arm pain. At investigation, a deep right breast lump was observed occupying the upper outer quadrant. Two palpable metastatic axillary nodes were observed during examination. The tumor was classified as a stage 2 loco‐regional malignancy, and the histopathology results confirmed the diagnosis of an invasive papillary carcinoma (grade 2), measuring 4 cm in maximum dimension. The patient underwent right mastectomy and axillary clearance.

### 3.4 Deleterious variants in *BRCA1* and *BRCA2* genes

The analysis of *BRCA1/2* genes of the 25 patients revealed two deleterious variants in two unrelated patients with total frequency of 8% (2/25), one within *BRCA1* and other within *BRCA2* (Table 1). The *BRCA1* variant c.4850C>A was nonsense mutation located in exon 16 and the *BRCA2* variant c.67+2T>C was a splice‐site mutation located in intron 2. As previously published, any type of insertion or deletion or amino acid substitution that result in premature stop codons before amino acids 1853 within the *BRCA1* was classified as mutated (Carraro et al., 2013). The identified heterozygous *BRCA1* nonsense variant c.4850C>A creates a premature stop codon at amino acid 1617 leading to premature truncated protein. The carrier of the *BRCA1* c.4850C>A which was detected in this study is an early onset patient, the age at onset was 33 years.

**Table 1:** *BRCA1* and *BRCA2* deleterious variants identified in the 25 breast cancer patients

Gene	Ex/Int	dbSNP	Genomic position	Nucleotide change	AA change	Mutation type	Var Allele Freq	BIC/ ClinVar	Clinical status
*BRCA1*	Ex. 16	—	Chr17:41223144	c.4850C>A	p.Ser1617X	NS	2%	Unreported/ Unreported	‐Invasive ductal carcinoma ‐Grade 3 ‐Stage 2B ‐FH (+)
*BRCA2*	Int. 2	rs81002885	Chr13:32890666	c.67+2T>C	Splice donor site	IVS Splice site	2%	CI/ Pathogenic	‐Invasive papillary carcinoma ‐Grade 2 ‐Stage 2B ‐FH (+)

Note

AA, amino acid; BIC, breast cancer core database; Cl, clinically significant; Ex, exon; FH, family history; Int, intron; IVS, intervening sequence; NS, nonsense; SNP, Single‐nucleotide polymorphism; (+), positive.

The second detected heterozygous *BRCA2* c.67+2T>C variant is located 2 nucleotide downstream of intron 2; The *BRCA2* c.67+2T>C splice‐site variant has been previously identified as a pathogenic mutation and already described in the BIC database (Diez et al., 2003; Houdayer et al., 2012).

### 3.5 Polymorphisms and unclassified sequence variants

Based on the data obtained by the NGS analysis, we have detected a total of 24 missense VUS including 11 *BRCA1* and 13 *BRCA2* variants (Tables 2 and 3). Of these, four *BRCA1* VUS and nine *BRCA2* VUS were rare variants according to 1000 genome project data (≤1% population minor allele frequency) (Table 2). By contract, seven *BRCA1* and four *BRCA2* were present at high frequencies in the study subjects and in >1% of the population (Table 3). According to 1000 genome project MAF data, those polymorphisms were indicated as common polymorphisms (Hirotsu et al., 2015; Landrum et al., 2014).

**Table 2:** Rare variants of uncertain significance found in patients with breast cancer

Gene	Ex/Int.	dbSNP	Genomic position	Nucleotide change	AA change	Mutation type	Var Allele Freq	BIC	ClinVar	SIFT prediction	1000 genome MAF (%)
*BRCA1*	Ex.11	rs56082113	Ch17:41245090	c.2458A>G	p.Lys820Glu	MS	2%	Unknown	Benign	TOLERATED	1.04
*BRCA1*	Ex.11	rs1800704	Ch17:41244524	c.3024G>A	p.Met1008Ile	MS	2%	Unknown	Benign	TOLERATED	0.06
*BRCA1*	Ex.11	rs2227945	Ch17:41244130	c.3418A>G	p.Ser1140Gly	MS	8%	Unknown	Benign	TOLERATED	1
*BRCA1*	Ex.16	rs1799967	Ch17:41222975	c.4956G>A	p.Met1652Ile	MS	2%	Unknown	Benign	TOLERATED	1
*BRCA2*	Ex.14	rs4986860	Ch13:32929309	c.7319A>G	p.His2440Arg	MS	2%	NCS	Benign	TOLERATED	1.04
*BRCA2*	Ex.22	rs4987047	Ch13:32953529	c.8830A>T	p.Ile2944Phe	MS	4%	NCS	Benign	**DAMAGING**	0.9
*BRCA2*	Ex.11	rs28897744	Ch13:32930673	c.7544C>T	p.Thr2515Ile	MS	2%	NCS	Benign	**DAMAGING**	—
*BRCA2*	Ex.11	rs41293485	Ch13:32912361	c.3869G>A	p.Cys1290Tyr	MS	2%	NCS	Benign	TOLERATED	0.34
*BRCA2*	Ex.11	rs4987117	Ch13:32914236	c.5744C>T	p.Thr1915Met	MS	2%	NCS	Benign	TOLERATED	0.8
*BRCA2*	Ex.11	rs35029074	Ch13:32914815	c.6323G>A	p.Arg2108His	MS	2%	Unknown	Benign	TOLERATED	0.38
*BRCA2*	Ex.24	rs80359189	Ch13:32954268	c.9242T>C	p.Val3081Ala	MS	2%	Unknown	Conflicting	**DAMAGING**	—
*BRCA2*	Ex.26	rs80359228	Ch13:32971119	c.9586A>G	p.Lys3196Glu	MS	2%	Unknown	Conflicting	**DAMAGING**	0.02
*BRCA2*	Ex. 27	rs11571833	Ch17:32972626	c.9976A>T	p.Lys3326*	NS	2%	NCS	Benign	N/A	0.4

Abbreviations: AA, amino acid; Ex, exon; Int, intron; MAF, minor allele frequency; MS, missense; NA, not available; NCS, No clinical significance; NS, nonsense; SIFT, sorting tolerant from intolerant; SNP, Single‐nucleotide polymorphism; Var, variant.

* Neutral stop codon.

**Table 3:** Common variants of uncertain significance found in patients with breast cancer

Gene	Ex/Int	dbSNP	Genomic position	Nucleotide change	AA change	Mutation type	Var Allele Freq	BIC	ClinVar	SIFT Prediction	1000 genome MAF (%)
*BRCA1*	Ex.11	rs16942	Ch17:41244000	c.3548A>G	p.Lys1183Arg	MS	38%	NCS	Benign	TOLERATED	35.260
*BRCA1*	Ex.11	rs16941	Ch17: 41244435	c.3113A>G	p.Glu1038Gly	MS	36%	NCS	Benign	**DAMAGING**	33.570
*BRCA1*	Ex.11	rs799917	Ch17: 41244936	c.2612C>T	p.Pro871Leu	MS	50%	NCS	Benign	**DAMAGING**	45.610
*BRCA1*	Ex.11	rs4986850	Ch17: 41245471	c.2077G>A	p.Asp693Asn	MS	10%	NCS	Benign	**DAMAGING**	3.360
*BRCA1*	Ex.16	rs1799966	Ch17: 41223094	c.4900A>G	p.Ser1634Gly	MS	38%	Unreported	Benign	**DAMAGING**	35.580
*BRCA1*	Ex.11	rs1799950	Ch17: 41246481	c.1067A>G	p.Gln356Arg	MS	2%	Unknown	Benign	**DAMAGING**	2.180
*BRCA1*	Ex.11	rs16940	Ch17: 41245237	c.2311T>C	p.Leu771Leu	MS	34%	NCS	Benign	NA	33.530
*BRCA2*	Ex.10	rs144848	Ch13: 32906729	c.1114A>C	p.Asn372His	MS	38%	NCS	Benign	TOLERATED	24.940
*BRCA2*	Ex.10	rs766173	Ch13: 2906480	c.865A>C	p.Asn289His	MS	6%	NCS	Benign	**DAMAGING**	7.370
*BRCA2*	Ex.14	rs169547	Ch13: 32929387	c.7397T>C	p.Val2466Ala	MS	96%	NCS	Benign	TOLERATED	2.42
*BRCA2*	Ex.11	rs1799944	Ch13: 32911463	c.2971A>G	p.Asn991Asp	MS	6%	NCS	Benign	TOLERATED	8.010

Abbreviations: AA, amino acid; Ex, exon; Int, intron; MAF, minor allele frequency; MS, missense; NA, not available; NCS, No clinical significance; SIFT, sorting tolerant from intolerant; SNP, Single‐nucleotide polymorphism; Var, variant.

We next applied SIFT (sorting tolerant from intolerant) which uses sequence homology to predict whether an amino acid substitution will affect protein function and hence, potentially alter phenotype to the missense substitutions associated with the disease. SIFT predicted 42% of the detected 24 variants to be damaging. The SIFT algorithm and software have been described previously (Ng & Henikoff, 2001, 2002).

## 4 DISCUSSION

The incidence of breast cancer is on the raise in Bahrain with a remarkable number of those affected are being diagnosed before they are 50 years old with advanced stage of cancer at presentation (Chouchane et al., 2013). The aggressive and early onset of the breast cancer in this population could be relatively explained by undermined breast cancer predisposition genetic factor(s) due to consanguineous marriages and shift in life styles (Hamadeh et al., 2014; Ravichandran & Al‐Zahrani, 2009). *BRCA1* and *BRCA2* germline mutations have been shown to play a significant role in genetic predisposition to breast cancer. To our knowledge, the contribution of *BRCA1* and *BRCA2* variants to hereditary breast cancer in Bahraini women has not been studied before. This is the first published report which shows the spectrum and the frequency of *BRCA1* and *BRCA2* variants among Bahraini women.

We performed a pilot whole *BRCA* gene sequencing study on DNA obtained from the peripheral blood samples of 25 Bahraini females with breast cancer. It was observed that the mean age of onset of breast cancer among this group of patients (47 years) is identical to the figures previously reported for the Arab population, a decade earlier than western countries (Fitzmaurice et al., 2015). Using a carefully optimized NGS screening approach, we were able to analyze all coding exons and flanking intronic regions of *BRCA1* and *BRCA2*. Based on documented knowledge on effects of variants that give rise to premature stop codons (via frameshift insertions or deletions, nonsense or consensus splice‐site sequence changes) or missense alterations at critical residues in functional domains, we defined one *BRCA1* c.4850C>A and one *BRCA2* c.67+2T>C as deleterious variants in two patients. Interestingly, both patients showed a very strong family history of breast cancer suggestive of genetic predisposition to breast cancer. The two deleterious variants were observed in two unrelated patients out of the 25 subjects studied providing an overall prevalence rate of 8%. Some studies conducted in other Arab countries such as Tunis and Saudi Arabia have reported frequencies of 16%–25% (Mahfoudh et al., 2012; Riahi et al., 2015) and 2.5%–28% (Merdad et al., 2015), respectively. Overall, as previous studies showed 3%–28% of familial breast cancer cases are linked to variants in *BRCA1* and *BRCA2* genes. The differences observed are mainly due to sampling size and other features related to patients inclusion criteria and their ethnicity.

In *BRCA1* gene, we detected one deleterious variant c.4850C>A in exon 16. In the present report, this variant was observed in a patient diagnosed with invasive ductal carcinoma at age 33. This patient reported to have a family history of breast cancer (a mother diagnosed with breast cancer at age 57, a daughter and maternal aunt diagnosed with breast cancer before age 40). *BRCA1* gene contains 22 exons spanning about 110 kb of DNA and encoding a 1863 amino acid protein with an N‐terminal RING finger domain and two BRCT—(*BRCA1*‐C‐terminal) domains. The identified *BRCA1* nonsense variant c.4850C>A was predicted to be causative because it creates a premature stop codon at amino acid 1617 leading to premature truncated protein. To the best of our knowledge, the *BRCA1* variant c.4850C>A was not reported previously elsewhere except in a recent study conducted in Qatar where two patients were found to be positive carriers for the c.4850C>A variant (Bujassoum et al., 2017). However, this variant has not been yet officially registered in the BIC database or other resources suggesting that it could be a unique or novel variant to the gulf region. Carraro et al. have detected the variant c.4968insGT in *BRCA1* for the first time in his study in early onset breast cancer Brazilian patients and had identified it as pathogenic deleterious variant because it results in a premature stop codon at amino acid 1617 (Carraro et al., 2013). This confirms our finding that the variant we identified in our study c.4850C>A is considered as deleterious variant as they both lead to a premature stop codon at amino acid 1617.

In *BRCA2* gene, we detected one splice‐site donor variant c.67+2T>C that leads to an aberrant transcript in intron 2. *BRCA2* is a large gene with 27 exons that encode a protein of 3,418 amino acids. The variant c.67+2T>C occurs within a consensus splice junction, and it is predicted to result in abnormal mRNA splicing which leads to unfunctional protein. The splice variants were generated by base substitutions, which either create or destroy splice acceptor and donor sites of the genes (Diez et al., 2003). In the present report, this variant was observed in a patient diagnosed with invasive ductal carcinoma whom was reported to have a strong family history of breast cancer (her mother, sisters, and maternal aunts). This variant is reported in the BIC database and had been found previously in Latin American/Western European populations (Diez et al., 2003).

Additionally, we have identified 24 sequence variants (11 *BRCA1* and 13 *BRCA2*) including distinct polymorphisms and unclassified sequence variants.

Among the identified SNPs, 5 *BRCA1* SNPs* (*rs1799950 (4%), rs16942 (64%), rs4986850 (20%), rs2227945 (16%), and rs1799966 (64%)) and 5 *BRCA*2 SNPs (rs766173 (12%), rs144848 (60%), rs4987117 (4%), rs4987047 (8%), and rs11571833 (4%)) are found to confer minor but cumulatively significant risk of breast cancer (Johnson et al., 2007). In cancer predisposition genes, there are 25 globally known SNPs identified to increase risk of breast cancer and of the 25, 14 SNPs are located in *BRCA1* and *BRCA2* genes (Johnson et al., 2007). Ten *BRCA* SNPs out of the 14 *BRCA* SNPs have been identified in our study subjects.

This study also confirmed the utility of NGS for performing the genetic testing of hereditary breast cancer based on *BRCA1* and *BRCA2* genetic alterations. As the causative mutations are distributed throughout the genes, we here recommend that that the technique is suitable to detect variants in *BRCA1* and *BRCA2* and other tumor suppressor genes. Identifying founder mutations would enable us to examine specific loci in the screening of high‐risk subpopulations for inherited breast cancer without performing a full sequence analysis of *BRCA1* and *BRCA2*. Founder mutations have previously been described for some population as in Ashkenazi Jewish population, 3% of individuals carried *BRCA1* c.185delAG, *BRCA1* c.5382insC or *BRCA2* c.6174delT mutations (Wiesman et al., 2017). The frequency of *BRCA1* and *BRCA2* mutations varies among population (Chopra & Kelly, 2017). The knowledge of the spectrum of mutations and their geographical distribution could provide more efficient approach for screening protocol and allow more rapid, less expensive and more affordable genetic testing strategy.

Our study had some limitations; the major one is the small sample size due to budget constraints and being a single institution study. The protocol applied here allows the identification of *BRCA* genes only; however, there are other few genes that were found to play a role in increasing susceptibility to breast cancer but at markedly lower frequency and penetrance. These genes include ATM, TP53, CHECK2, PTEN, STK11, PALB2, BRIP, and the RAD51 genes (Merdad et al., 2015). In fact, the frequency of TP53 mutations among Saudi patients is one of the highest in the world (Al‐Qasem et al., 2011; Chopra & Kelly, 2017). Further researches are needed to elucidate the spectrum of mutations in those genes to confirm their association with the increase risk of developing breast cancer in the population.

## 5 CONCLUSION

In conclusion, this is the first report on the breast cancer predisposition factors in the population of Bahrain and our data suggest that *BRCA1* and *BRCA2* variants may contribute to the pathogenesis of familial breast cancer in Bahraini patients. We detected two deleterious *BRCA1* and *BRCA2* variants in two unrelated patients, one in *BRCA1* gene (c.4850C>A) and other in *BRCA2* gene (c.67+2T>C). The identified *BRCA1* variant c.4850C>A has been reported in a Qatari study and in this report only suggesting that it might be novel to the gulf region. We also showed that NGS is a useful tool to explore mutations in *BRCA1* and *BRCA2* genes and can be used to screen mutations in probands and unaffected relatives from the same family.

## CONFLICT OF INTEREST

The authors declare that there is no conflict of interest regarding the publication of this article.

## AUTHOR'S CONTRIBUTIONS

FA conceived the study, participated in its design and coordination, carried out the molecular genetics studies, analyzed the data, and drafted the manuscript. MK arranged for the study funding, participated in the study coordination, and revised the manuscript. ST supervised the sequencing experiment and revised the manuscript. LA looked after patients' recruitment, gathering the clinical data, and revised the manuscript. All the authors read and approved the final manuscript.

## Data Availability

All the data used in this study are from public sources. The reference sequences (accession numbers: NM_007294.3 for *BRCA1* and NM_000059.3 for *BRCA2*) used in this study were obtained from the National Center for Biotechnology Information sequence read archive, NCBI (https://www.ncbi.nlm.nih.gov/), and is publicly available for non‐commercial purposes. The clinical significance of each sequence variant is suggested based on reference to ClinVar and BIC. BIC: Breast Cancer Information Core (http://research.nhgri.nih.gov/bic). ClinVar: Database of mutations and their clinical relevance (http://www.ncbi.nlm.nih.gov/clinvar/). The NGS datasets generated during the current study are available from the corresponding author (in FASTQ files) on reasonable request.
